# Innate immune signalling, neuroinflammation and network plasticity in temporal lobe epilepsy

**DOI:** 10.3389/fphar.2026.1770964

**Published:** 2026-02-11

**Authors:** Oscar Arias-Carrión, Julieta Rodríguez de Ita, Philipp Yu

**Affiliations:** 1 División de Neurociencias Clínica, Instituto Nacional de Rehabilitación Luis Guillermo Ibarra Ibarra, Mexico City, Mexico; 2 Tecnologico de Monterrey, Escuela de Medicina y Ciencias de la Salud, Mexico City, Mexico; 3 Tecnologico de Monterrey, Escuela de Medicina y Ciencias de la Salud, Monterrey, Mexico; 4 Institute of Immunology, Philipps-Universität Marburg, Marburg, Germany

**Keywords:** blood–brain barrier, epilepsy, epileptogenesis, HMGB1, immunotherapy, inflammasome, interleukin-1β, microglia

## Abstract

Temporal lobe epilepsy emerges from a cascade of molecular, cellular, and structural disturbances triggered by heterogeneous cerebral insults—including convulsive status epilepticus, viral encephalitis, traumatic brain injury, and blood–brain barrier disruption—that converge on progressive hippocampal reorganization and a chronic predisposition to unprovoked focal seizures. Convergent evidence from chemoconvulsant models, focal intrahippocampal kainate administration, viral encephalitis paradigms, organotypic hippocampal cultures, human iPSC-derived organoids, and resected human tissue shows that innate immune pathways are not secondary epiphenomena but central drivers of epileptogenesis. Pattern-recognition receptors—particularly TLR2, TLR3, TLR4, IL-1R1 and the NLRP3 inflammasome—sense pathogen- and damage-associated molecular motifs, including HMGB1, and initiate MyD88-, NF-κB- and caspase-1–dependent signaling. These cascades acutely amplify IL-1β, TNF-α and IL-6 responses, alter ion-channel phosphorylation states, enhance NMDA- and AMPA-receptor–mediated excitation, and impair GABAergic inhibition, thereby lowering the seizure threshold. Sustained innate immune activation drives microglial M1 polarization, complement-mediated synaptic loss, aberrant neurogenesis, endothelial dysfunction, and persistent astroglial reactivity—mechanisms that reinforce circuit hyperexcitability and enable the transition from provoked to spontaneous recurrent seizures. Targeted interventions—including TLR4 antagonists (TAK-242), IL-1–pathway inhibitors (anakinra; the caspase-1 inhibitor VX-765), NLRP3 inhibitors (MCC950), and complement-directed strategies—reduce seizure burden, mitigate hippocampal atrophy, and, when administered early, attenuate maladaptive network remodeling. Several conventional antiseizure medications, including levetiracetam, also exhibit immunomodulatory properties by modulating microglial activation, suggesting a mechanistic overlap between pharmacological seizure control and immune regulation. Emerging data implicate the TLR7–endogenous retrovirus axis as an upstream determinant of neuroimmune homeostasis, linking impaired surveillance of viral and retroelement activity to glial activation and network instability. Together, these findings position innate immunity as a mechanistically coherent and therapeutically tractable axis in temporal lobe epilepsy. Achieving clinical translation will require immune-phenotype stratification, biomarker-guided timing of intervention, and advances in CNS-targeted delivery. Integrating immunomodulatory approaches with established antiseizure therapies offers a promising route toward disease modification, cognitive preservation, and more precise treatment of drug-resistant epilepsy.

## 1 Introduction

Epilepsy affects more than 50 million people globally and remains one of the most disabling neurological disorders (Secco, 2020). Defined by recurrent unprovoked seizures, epilepsy compromises cognition, memory, and quality of life, while imposing a high burden of psychiatric and systemic comorbidities (Devinsky et al., 2018). Recent studies demonstrate that chronic temporal lobe epilepsy (TLE) is associated with measurable impairments in episodic memory, executive function, and attentional processing, reflecting structural and inflammatory alterations within the hippocampal network. Among focal epilepsies, TLE is the most prevalent and the most treatment-resistant subtype, accounting for nearly 60% of drug-resistant cases (Engel, 2016; Pitkanen et al., 2021). Although precipitating factors such as traumatic brain injury, prolonged febrile seizures, or central nervous system (CNS) infection are well documented, aetiology remains unexplained in many patients (Verellen and Cavazos, 2010; Golub and Reddy, 2022). This clinical and biological heterogeneity underscores the need to define the molecular mechanisms that initiate epileptogenesis and sustain chronic seizure circuitry.

The long-standing view that the CNS is immunologically privileged has been fundamentally revised (Louveau et al., 2015). It is now clear that the brain is an active immune environment capable of rapid, context-dependent responses to injury, infection, metabolic stress, and aberrant neuronal activity (Li et al., 2023; Vezzani et al., 2023). Within this framework, neuroinflammation is increasingly recognized not only as a consequence of seizures but as a mechanistic contributor to disease initiation and progression (Sanz et al., 2024). This is particularly evident in conditions marked by acute tissue injury, blood–brain barrier (BBB) disruption, or sustained activation of resident glial populations (Zhang et al., 2023).

Histopathological analyses of resected hippocampi from patients with TLE reveal widespread microgliosis, astrogliosis, cytokine upregulation, and immune cell infiltration—even in the absence of infectious or autoimmune pathology (Iori et al., 2016; Terreros-Roncal et al., 2021). Advanced neuroimaging corroborates these findings, linking microglial activation with hippocampal atrophy and cognitive decline (Towne et al., 2025). Experimental models similarly demonstrate an early wave of glial activation, BBB dysfunction, and cytokine release preceding the development of spontaneous recurrent seizures (Kirkman et al., 2010; West et al., 2022). Emerging evidence also suggests that peripheral immune signals—including gut-derived metabolites and systemic inflammatory mediators—can influence central excitability and microglial phenotype, indicating a broader network of immune–neural interactions (Balakrishnan et al., 2024).

Mechanistic studies have delineated specific immune pathways that modulate neuronal excitability and reshape synaptic architecture. Interleukin-1β (IL-1β) enhances N-methyl-D-aspartate receptor (NMDAR) function via a sphingomyelinase–Src–NR2B signalling cascade (Chen et al., 2021). Danger-associated molecular patterns (DAMPs), particularly high-mobility group box 1 (HMGB1), activate Toll-like receptor 4 (TLR4) and the receptor for advanced glycation end-products (RAGE), altering synaptic plasticity and BBB integrity (Iori et al., 2016; Ping et al., 2021). The NLRP3 inflammasome amplifies these processes, and pharmacological inhibition reduces seizure burden, preserves cognition, and shifts microglial populations toward reparative states (El-Sayed et al., 2023; Hong et al., 2024; Wang et al., 2024; Fawzy et al., 2025). Complement activation, particularly C3 upregulation, contributes to aberrant synaptic pruning and vascular dysfunction (Aronica et al., 2007). Single-cell transcriptomics and spatial proteomics further reveal disease-associated glial states that correlate strongly with seizure severity (Kumar et al., 2022; Piwecka et al., 2023). In addition to pro-inflammatory programs, glial cells exhibit context-dependent neuroprotective phenotypes—including anti-inflammatory cytokine signalling and trophic factor release—although these compensatory responses appear blunted or dysregulated in chronic TLE (Zhang et al., 2025). Together, these insights establish neuroinflammation as a central mechanism that shapes both acute hyperexcitability and long-term circuit remodeling, driving the transition from an initial insult to chronic epilepsy.

In this review, we synthesize evidence from animal models and human studies to illustrate how innate immune sensors—including IL-1β, HMGB1–TLR4/RAGE signalling, complement activation, and the TLR7–endogenous retrovirus (ERV) axis—govern seizure susceptibility and network reorganization. We further examine emerging immunomodulatory strategies, identify barriers to translation, including timing, specificity, and BBB penetration, and outline a precision framework integrating immune-targeted therapies with established antiseizure medications to modify the disease trajectory in drug-resistant TLE.

## 2 Animal models of temporal lobe epilepsy

### 2.1 Inflammatory signalling as a driver of seizure susceptibility

Experimental models of TLE have been indispensable in delineating how neuroinflammatory cascades shape seizure initiation and progression (Rusina et al., 2021). Established paradigms—including pilocarpine- and kainate-induced status epilepticus (SE) and viral encephalitis—reproduce key features of mesial TLE, such as hippocampal sclerosis, spontaneous recurrent seizures, and cognitive decline (Table 1). Complementary approaches, such as organotypic slice cultures and targeted inflammatory sensitization, enable a more detailed dissection of upstream molecular mechanisms (Magalhaes et al., 2018). Comparative analyses demonstrate that these models differ meaningfully in their inflammatory signatures—for example, kainate exposure elicits robust IL-1β and TLR4 activation, whereas pilocarpine produces stronger NLRP3 engagement and oxidative stress, and viral encephalitis predominantly induces TNF-α and IL-6 responses (El-Sayed et al., 2023; Fawzy et al., 2025). Collectively, these models have clarified how immune mediators alter excitability and network dynamics, revealing model-specific immune pathways that inform therapeutic targeting strategies (Vezzani et al., 2023).

**TABLE 1:** Innate immune pathways and mechanistic insights across experimental and human models of temporal lobe epilepsy.

Study	Targeted Immune Pathway	Model System	Species	Key Mechanistic Insight	Therapeutic Implication
Alexopoulou et al. (2001)	TLR3 → type-I IFN signalling	Poly (I:C)-induced seizures	Mouse	TLR3 activation increases IFN-1 and excitability; IL-1β-independent	TLR3 inhibition may prevent infection-linked ictogenesis
Balosso et al. (2008)	IL-1β → IL-1R1 → sphingomyelinase/Src/NR2B	Systemic kainate (acute)	Mouse	Rapid non-transcriptional NR2B phosphorylation → hyperexcitability	IL-1R1 or Src pathway inhibitors reduce acute seizures
Bernardino et al. (2008)	P2X7-dependent IL-1β release	Organotypic hippocampal slice cultures	Rat	Microglial P2X7 activation triggers IL-1β release and AMPA excitotoxicity	P2X7 blockade or IL-1β antagonism prevents neuron loss
Maroso et al. (2010)	HMGB1 → TLR4	Intrahippocampal kainate	Mouse	HMGB1–TLR4 engagement enhances NR2B phosphorylation and prolongs seizures	TLR4 antagonism reduces acute and spontaneous seizures
Iori et al. (2013)	HMGB1 → TLR4 & RAGE	Chronic IHpKA; human mTLE	Mouse/Human	Dual receptor engagement drives gliosis, BBB disruption, and altered neurogenesis	Receptor-selective inhibition may protect the BBB and synapses
Noe et al. (2013)	IL-1R1 blockade/caspase-1 inhibition	Pilocarpine SE; electrical SE	Rat	Delayed blockade reduces IL-1β and neurodegeneration but not spontaneous recurrent seizures	Time-sensitive IL-1 pathway targeting is needed for disease modification
Kirkman et al. (2010)	TNF-α & IL-6 (MyD88-independent)	TMEV viral encephalitis	Mouse	TNF-α/IL-6 essential for seizures; IL-1R1/MyD88 not required	Cytokine-specific targeting in infection-driven epilepsy
Aronica et al. (2007)	Complement C3 activation	Human TLE tissue + rodent SE	Human/Rodent	C3 upregulation → gliosis, synaptic pruning, BBB dysfunction	C3a/C5a inhibitors may protect synapses and BBB integrity
Itoh et al. (2019)	Microglial modulation by levetiracetam	Chemoconvulsant SE	Rat	Levetiracetam reduces microglial activation and IL-6	ASD choice may leverage anti-inflammatory properties
Dong et al. (2022)	TLR4 → NF-κB (TAK-242)	Kainate-induced epilepsy	Mouse	TLR4 blockade preserves the BBB and lowers IL-1β/TNF-α	TAK-242 demonstrates disease-modifying potential
Ping et al. (2021)	HMGB1–TLR4 signalling	KA & PILO models	Mouse	Confirms HMGB1–TLR4-driven hyperexcitability and BBB injury	Supports HMGB1–TLR4 targeting in TLE
Cristina de Brito Toscano et al. (2021)	NLRP3 inflammasome	Human mTLE hippocampus	Human	Elevated NLRP3 & active caspase-1 in sclerotic hippocampi	Validates NLRP3 as a translational target
Wang et al. (2024)	NLRP3 inhibition (MCC950)	Pilocarpine-induced epileptogenesis	Mouse	MCC950 reduces M1 microglia, seizures, and preserves cognition	Strong candidate for disease modification
Solanki and Jha (2025)	Anti-inflammatory IL-10 & Treg pathways	Autoimmune epilepsy models	Mouse	IL-10/Treg dysfunction worsens excitability and neuroinflammation	Immunomodulation should preserve regulatory immune tone
Victor and Tsirka (2020)	Microglial suppression (minocycline)	Chemoconvulsant SE	Mouse	Reduces IL-6, ROS, and aberrant neurogenesis	Broad-spectrum microglial modulation with clinical potential
Geng et al. (2019)	B cell/antibody deficiency	Human TLE	Human	Subsets of TLE patients show immune dysregulation and low protective antibodies	Supports stratifying patients by B cell/antibody phenotype
Lee et al. (2023)	Nanoparticle drug delivery	Preclinical BBB models	Rodent	Nanocarriers improve CNS penetration of immunomodulators	Enhances the feasibility of targeted immunotherapies
Fawzy et al. (2025)	TLR4–NLRP3 inflammasome signalling in hippocampal sclerosis	Chronic TLE with hippocampal sclerosis	Human tissue + mouse	TLR4-driven NLRP3 activation correlates with neuronal loss and network hyperexcitability in HS-TLE	Supports dual targeting of TLR4–NLRP3 in HS-associated drug-resistant TLE
El-Sayed et al. (2023)	Microglial NLRP3/IL-1β axis → BBB dysfunction	Kainate-induced chronic TLE	Mouse	Microglial NLRP3 activation promotes BBB leakage and seizure susceptibility	NLRP3 inhibition and BBB-stabilizing agents show synergistic protection

Across chemoconvulsant SE, focal intrahippocampal kainate, viral encephalitis, organotypic slices, human iPSC-derived systems, and resected human tissue, innate immune cascades—TLR4, IL-1β, complement C3, and the NLRP3 inflammasome—consistently modulate excitability, BBB, integrity, and maladaptive circuit remodeling. The two newly added studies further strengthen the evidence that TLR4–NLRP3 coupling and microglia-driven BBB, failure are central to hippocampal sclerosis and chronic TLE., early, phase-specific interventions—TLR4, antagonists, inflammasome inhibitors; IL-1, pathway modulators, complement blockade—remain the most compelling translational candidates, especially when paired with biomarkers for inflammatory phenotyping and nanocarrier-enhanced CNS, delivery.

### 2.2 Cytokine signalling and acute hyperexcitability

Seminal work with systemic kainate exposure demonstrated that IL-1β rapidly increases seizure severity through a non-transcriptional cascade involving sphingomyelinase-dependent ceramide release, Src kinase activation, and NR2B phosphorylation on NMDA receptors (Balosso et al., 2008). Inhibition of this pathway suppressed ictogenesis, defining a clinically relevant molecular axis independent of gene transcription. While this model does not recapitulate chronic epileptogenesis, it provided early evidence that cytokines can acutely modulate neuronal excitability.

*Ex vivo* organotypic hippocampal slice cultures extended these insights by showing that microglial activation via the P2X7 receptor, in response to LPS and ATP, triggers IL-1β release, sensitizing neurons to AMPA receptor–mediated excitotoxicity. Pharmacological blockade of P2X7 or IL-1β prevented neuronal death, highlighting a microglia–neuron signalling axis critical for hippocampal vulnerability (Bernardino et al., 2008). These findings also illustrate the rapidity with which cytokine signalling modifies ionotropic receptor dynamics, reshaping synaptic integration within minutes to hours.

### 2.3 Viral encephalitis and innate immunity

In Theiler’s murine encephalomyelitis virus (TMEV) infection, TNF-α and IL-6 were identified as essential drivers of limbic seizures, whereas IL-1β and MyD88 signalling were dispensable (Kirkman et al., 2010). This context-specificity illustrates that innate immune pathways contribute differentially to ictogenesis depending on the nature of the initiating insult. Recent viral models, including West Nile virus and Zika virus, have also implicated cytokine-driven disruption of hippocampal circuitry, underscoring infection as a clinically relevant trigger of epileptogenesis (Stewart et al., 2010; Lourenco et al., 2025). These models further highlight that distinct viral pathogens engage unique receptor pathways—such as TLR3 for double-stranded RNA viruses—producing seizure phenotypes that reflect pathogen-specific host responses rather than a uniform inflammatory signature.

### 2.4 Therapeutic implications of cytokine modulation

In rat models of SE, delayed administration of anakinra (IL-1 receptor antagonist) or VX-765 (caspase-1 inhibitor) reduced astroglial IL-1β expression and hippocampal neurodegeneration, but failed to alter long-term seizure frequency (Noe et al., 2013). These findings suggest that late intervention may confer neuroprotection without altering chronic epileptogenesis, thereby reinforcing the principle that therapeutic timing is crucial. Early intervention—within the initial post-insult immunological window—appears necessary to influence the progression toward chronic TLE, as supported by recent data demonstrating reduced seizure frequency and preserved cognitive performance following timely blockade of IL-1R1 or NLRP3 activation (El-Sayed et al., 2023; Fawzy et al., 2025).

### 2.5 DAMP–receptor interactions in epileptogenesis

DAMPs such as HMGB1 are released from injured neurons and reactive glia during seizures. HMGB1 binding to TLR4 enhances NMDA receptor phosphorylation and prolongs seizure duration; genetic deletion or pharmacological inhibition of TLR4 reduces both acute and spontaneous seizures (Maroso et al., 2010; Zhang et al., 2022). Parallel work demonstrates that HMGB1 also signals through the receptor for advanced glycation end products (RAGE), which is selectively upregulated in human TLE and in experimental models. In RAGE-deficient mice, seizure duration is shortened, but the downstream effects on aberrant neurogenesis and hippocampal remodeling differ from those observed in TLR4-deficient mice, indicating receptor-specific contributions to epileptogenesis and circuit pathology (Iori et al., 2016). Recent studies confirm that HMGB1–TLR4 blockade not only reduces seizure susceptibility but also preserves synaptic integrity and memory performance, supporting a strong mechanistic link between DAMP signalling and cognitive decline (Zhang et al., 2022).

### 2.6 Emerging roles of innate immune receptors

Beyond TLR4 and RAGE, additional innate immune sensors may contribute to shaping epileptogenesis. The three endosomal Toll-like receptors—TLR3 (dsRNA), TLR7 (ssRNA), and TLR9 (CpG DNA)—share nucleic acids as their natural ligands. At present, it is unclear whether endogenous nucleic acid–derived DAMPs or persistent viral sequences activate these receptors within TLE-affected hippocampal circuits. Differences in cytokine profiles across models suggest that endosomal TLR activation may be injury-specific rather than universally engaged.

Chronic viral infections offer a plausible link: impaired TLR3 function predisposes to herpes simplex encephalitis and may contribute to febrile infection–related epileptic syndrome (FIRES) (Hsieh et al., 2020; Zhang and Casanova, 2024). TLR7 and TLR9 dysfunction has also been implicated, raising the possibility that coordinated endosomal signalling deficits influence susceptibility to post-infectious epilepsy. Moreover, recent work suggests that dysregulated TLR7 activity could permit accumulation of endogenous retroviral RNA, potentially lowering seizure threshold through sustained microglial activation—a hypothesis explored later in this review.

Complement cascade activation, particularly C3 upregulation, has also been implicated in BBB dysfunction, gliosis, and synaptic pruning in both animal models and patient-derived tissue (Aronica et al., 2007; Terreros-Roncal et al., 2021). This further supports the concept that innate immune receptors and downstream effector pathways converge to reshape hippocampal networks.

### 2.7 Antiseizure drugs and immunomodulation

Accumulating evidence suggests that classical antiseizure drugs (ASDs) possess immunomodulatory properties. Sodium channel blockers such as carbamazepine and vinpocetine suppress hippocampal IL-1β and TNF-α expression even under conditions of inflammatory challenge, whereas valproate lacks this effect (Sitges et al., 2014). More recent studies show that levetiracetam attenuates microglial activation and reduces IL-6 production, suggesting that anti-inflammatory capacity may contribute to the clinical efficacy of specific ASDs (Itoh et al., 2019; Matsuo et al., 2022). These observations indicate that immunomodulatory effects of ASDs may complement their electrophysiological actions, offering an opportunity for rational combination therapy.

### 2.8 Inflammasome signalling as a therapeutic target

The NLRP3 inflammasome has emerged as a critical mediator of chronic neuroinflammation (El-Sayed et al., 2023). In pilocarpine-induced SE, pharmacological inhibition with MCC950 attenuated microglial M1 polarization, reduced hippocampal IL-1β levels, and improved seizure burden and cognitive outcomes (Wang et al., 2024; Fawzy et al., 2025). Complementary evidence from human epileptic tissue confirms upregulation of NLRP3 and caspase-1, supporting its translational relevance (Cristina de Brito Toscano et al., 2021). Given the conserved role of inflammasome activation across injury models, targeting NLRP3 offers a unifying strategy to modulate both acute ictogenesis and chronic network remodeling (El-Sayed et al., 2023).

### 2.9 Translational outlook

Taken together, these models converge on a framework in which brain inflammation—whether triggered by excitotoxicity, viral infection, or trauma—activates defined cytokine–receptor axes that amplify excitability, propagate seizures, and remodel hippocampal circuits. Central to these processes are the IL-1β–sphingomyelinase–Src–NR2B pathway, NLRP3 inflammasome activation, HMGB1–TLR4 and HMGB1–RAGE signalling, and pro-inflammatory cytokines such as TNF-α and IL-6 (El-Sayed et al., 2023).

By clarifying how these immune pathways regulate excitability, validated animal models provide a rational foundation for immunomodulatory interventions that aim to move beyond symptomatic seizure suppression toward disease modification in pharmacoresistant TLE. Future advances will depend on integrating immune biomarkers into preclinical design, refining timing and dosing strategies, and developing delivery systems that achieve CNS-selective immune modulation while preserving essential homeostatic functions.

## 3 The role of the immune system in epilepsy

For much of the 20th century, the immune system was considered peripheral to brain physiology. This view has shifted decisively: the immune system is now recognized as an essential regulator of neural homeostasis, plasticity, and pathology (Passaro et al., 2021). This paradigm shift has transformed our understanding of epilepsy. The CNS, once thought to be immunologically privileged, is now understood as a specialized immune environment capable of dynamic, context-dependent responses to injury, infection, and aberrant neuronal activity (Vezzani et al., 2019; Passaro et al., 2021; Vezzani et al., 2023). A recent study proposed an alternative to the now-debunked immune-privilege model: at the border of the brain parenchyma, glial (glymphatic) pathways and meningeal lymphatics facilitate active immunosurveillance while limiting aberrant immune responses (Kim and Kipnis, 2025). Whether this relatively new concept is instrumental to our understanding of epilepsy needs to be further examined. Notably, neuroinflammation is increasingly recognized as a mechanistic driver of both seizure initiation and long-term circuit remodeling, linking diverse epilepsy subtypes through convergent immune pathways. Evidence from acquired, genetic, and autoimmune epilepsies suggests that neuroinflammation represents a convergent pathophysiological mechanism—and a tractable therapeutic target.

### 3.1 Innate immunity: the first responder and its RNA-metabolism control

**FIGURE 1:**
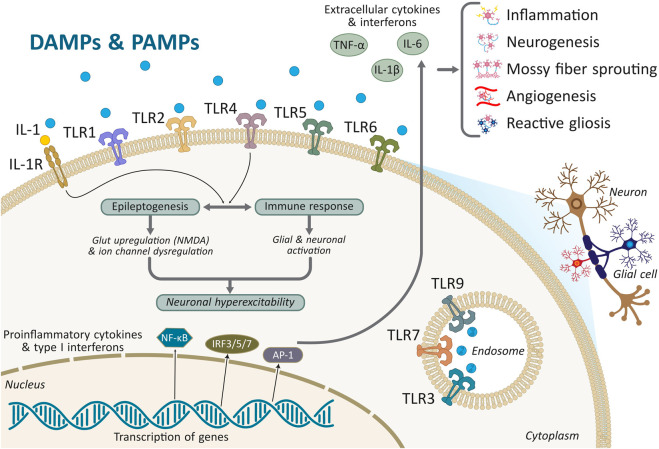
Molecular architecture of innate immune activation driving epileptogenesis. Damage- and pathogen-associated molecular patterns (DAMPs and PAMPs) engage pattern-recognition receptors at the neuronal and glial surface—including IL-1R1 and Toll-like receptors (TLR1/2/4/5/6)—as well as endosomal TLRs (TLR3/7/9). Ligand binding initiates MyD88-and TRIF-dependent signalling that rapidly activates NF-κB, IRF3/5/7, and AP-1, leading to the transcription of proinflammatory cytokines and type I interferons. IL-1R1– and TLR4-dependent pathways potentiate glutamate receptor (e.g., NMDA) phosphorylation, dysregulate ion channel activity, and amplify glial–neuronal crosstalk, thereby increasing neuronal hyperexcitability. Extracellular cytokines (IL-1β, TNF-α, IL-6) and interferons reinforce immune activation, glial recruitment, and metabolic stress, establishing self-perpetuating inflammatory loops. Chronic signalling disrupts blood–brain barrier integrity, alters astrocytic and microglial states, and drives structural remodeling, including aberrant neurogenesis, mossy fibre sprouting, angiogenesis, and reactive gliosis—changes that collectively destabilize hippocampal circuits and promote seizure generation. The figure highlights how convergent IIS pathways integrate molecular triggers with structural outcomes, underscoring the therapeutic potential of targeting IL-1R1, TLR4, and downstream inflammasome signalling (e.g., NLRP3) to modify epileptogenesis.

Innate immunity constitutes the first line of defence against CNS insults and plays a pivotal role in early epileptogenesis (Figure 1). Microglia, the brain’s resident immune cells, express pattern-recognition receptors such as TLRs that detect both pathogen-associated and endogenous danger signals. Among these, TLR4 activation by HMGB1 increases excitability by phosphorylating NR2B on NMDA receptors, accelerating seizure onset (Maroso et al., 2010). In rodent TLE models, HMGB1–TLR4 and HMGB1–RAGE signalling prolong seizures, alter hippocampal neurogenesis, and destabilize synaptic integrity (Iori et al., 2016; Zaben et al., 2021; Li et al., 2023; Dahalia et al., 2024). These receptor-specific signalling axes demonstrate that discrete DAMP-mediated pathways can selectively influence excitability, plasticity, and memory-associated networks.

IL-1β, released from activated glia during SE, engages a sphingomyelinase–Src–NR2B cascade to increase excitatory drive independently of gene transcription (Balosso et al., 2008). Pharmacological inhibition of this pathway reduces seizure severity within minutes, underscoring its clinical relevance. *Ex vivo* hippocampal slice cultures complement this evidence: P2X7 receptor activation triggers IL-1β release, sensitizing neurons to AMPA receptor-mediated excitotoxicity; blockade of P2X7 or IL-1β prevents cell death, highlighting a microglia–neuron excitability axis (Bernardino et al., 2008). These findings collectively position IL-1β signalling as a rapid-acting modulator of synaptic integration and as a potential early therapeutic target.

A critical intracellular amplifier of innate immunity is the NLRP3 inflammasome, which integrates upstream TLR signals to drive caspase-1–mediated IL-1β maturation (El-Sayed et al., 2023). In pilocarpine-induced SE, pharmacological inhibition with MCC950 reduced seizure burden, lowered hippocampal IL-1β levels, shifted microglial phenotypes toward M2-like states, and preserved cognition (Wang et al., 2024). Human studies confirm increased NLRP3 expression in resected epileptic hippocampi (Pohlentz et al., 2022), validating its translational potential. Thus, inflammasome activation represents a nodal point at which multiple danger signals converge, amplifying neuroinflammatory tone and promoting circuit instability.

Viral models provide further nuance. In Theiler’s murine encephalomyelitis virus infection, seizures depend on TNF-α and IL-6 but not IL-1β, demonstrating that innate pathways vary with the initiating insult (Kirkman et al., 2010). These differential cytokine dependencies underscore the context-specificity of innate immune activation and support a model in which distinct inflammatory profiles shape unique epileptogenic trajectories. Together, these findings position innate immunity as the central driver of early ictogenesis.

In recent years, the field of RNA biology has made substantial advances. In particular, the question of how RNA metabolism in activated immune cells is controlled is now better understood. It is clear that, in cells of the immune system, after the initial activation through TLRs, NLRs, cGAS, or C-type lectins (CLRs), cellular programs are triggered that play a pivotal role in the transcriptional activation of pro-inflammatory cytokines, e.g., IL-6. A complex mRNA-binding and degrading machinery is engaged in various cleavage processes that reduce, e.g., IL-6 mRNA levels. The best-studied RNA-binding proteins are Roquin-1/2, also known as Regnase-related endonucleases (Yoshinaga and Takeuchi, 2024), which are active in microglia (Liu et al., 2016). Disruption of these RNA-regulatory programs may prolong inflammatory signalling and lower seizure threshold, suggesting that targeting RNA-metabolism pathways could represent a new therapeutic Frontier in neuroimmunology. The development of drugs targeting the function of innate RNA metabolic players could lead to a new class of anti-inflammatory medications that could also benefit complex CNS diseases like TLE.

### 3.2 Adaptive immunity: chronic inflammation and CAR-T–related neuroinflammation linked to B cell dysfunction

Although slower to engage, adaptive immunity sustains chronic neuroinflammation, particularly once BBB disruption permits immune infiltration. CD8^+^ T cells induce neuronal apoptosis, while CD4^+^ helper T cells amplify glial activation through IFN-γ and IL-17 (Alvarado and Brewster, 2021). Impaired regulatory T cell (Treg) function skews the balance toward pro-inflammatory states (Yue et al., 2022). These adaptive mechanisms contribute to prolonged circuit instability and may influence drug responsiveness in chronic TLE. Autoimmune epilepsies—such as anti-NMDA receptor encephalitis—illustrate the pathogenic potential of adaptive immunity. Autoantibodies against neuronal surface antigens cause synaptic dysfunction and seizures but respond to corticosteroids, IVIG, or monoclonal antibodies, highlighting the therapeutic value of immune modulation (Dalmau et al., 2017; Solanki and Jha, 2025).

In recent years, the new CAR-T cell therapy technique has entered the clinic (Ortuno-Sahagun et al., 2025). Worldwide, an increasing number of patients with leukaemia and B-cell-mediated autoimmunity have been treated with B-cell-specific CAR-T cells (Muller et al., 2024). However, in a substantial percentage of treated patients, an until-then unknown syndrome developed—immune effector cell-associated neurotoxicity syndrome (ICANS). Acute seizures and SE are observed in patients treated with B cell lymphoma (Saw et al., 2022; Pensato et al., 2024). Interestingly, the target antigen CD19 of CAR-T cells is restricted to B cells and is not expressed by CNS cells. Despite this, emerging data indicate that CAR-T treatment induces widespread microglial activation and white-matter inflammation, suggesting that systemic immune perturbation alone can precipitate CNS hyperexcitability.

The epileptic phenotype in CAR-T-treated patients may also reflect a second mechanism: reduced production of “anti-inflammatory” antibodies. Antibody deficiency has been reported in a subset of individuals with TLE (Geng et al., 2019). Loss of protective immunoglobulin repertoires may remove a homeostatic buffer against inflammation-driven hyperexcitability, providing a mechanistic bridge between CAR-T–induced neurotoxicity and familial TLE. This notion aligns with the moderate efficacy of IVIG therapy in drug-resistant epilepsy (Doran et al., 2025).

In contrast, in non-autoimmune TLE, adaptive responses appear secondary. In TMEV infection, seizures developed independently of virus-specific T cells, suggesting that innate rather than adaptive mechanisms are dominant ictogenic drivers (Kirkman et al., 2010). Nonetheless, emerging data show clonal T cell expansions and persistent antibody responses in subsets of patients with drug-resistant epilepsy (Hendrix et al., 2024; Mu et al., 2025). These findings indicate that adaptive immunity contributes to chronicity in selected patients, particularly those with persistent immune activation or subtle BBB dysfunction.

### 3.3 Innate–adaptive crosstalk in epileptogenesis

Crosstalk between innate and adaptive immunity orchestrates the transition from acute to chronic inflammation. Microglia and astrocytes, acting as antigen-presenting cells, prime T cells and shape the cytokine milieu. In turn, lymphocyte-derived cytokines sustain glial reactivity, creating a self-perpetuating inflammatory loop. TLR3 and TLR4 emerge as central hubs that mediate dendritic-cell recruitment, immune activation, and cytokine release (Moresco et al., 2011; Piwecka et al., 2023). This bidirectional communication ensures that even limited immune infiltration can sustain prolonged neuroinflammation, thereby coupling innate danger signals to adaptive immune persistence. Even in acquired TLE, where immune infiltration is limited, neuroinflammation persists due to enduring interactions between glia and the immune system (Terreros-Roncal et al., 2021). Thus, immune dysregulation is not simply a by-product of seizures but a core driver of epileptogenesis.

### 3.4 Therapeutic implications and future directions

This evolving neuroimmune framework provides a robust rationale for therapeutic innovation. In preclinical models: a) IL-1β inhibitors (e.g., anakinra) reduce neuronal death and seizure severity (Noe et al., 2013). b) NLRP3 antagonists (e.g., MCC950) lower seizure burden and protect cognition (Wang et al., 2024; Fawzy et al., 2025). c) TLR4 antagonists (e.g., TAK-242) dampen HMGB1-driven hyperexcitability and preserve BBB function (Dong et al., 2022).

Classical ASDs also exert immunomodulatory effects; carbamazepine and valproate reduce hippocampal IL-1β and TNF-α, whereas vinpocetine lacks this property (Sitges et al., 2014). The differential impact of ASDs on inflammatory pathways suggests that immunomodulatory capacity may contribute to their clinical efficacy, especially in drug-resistant epilepsy. Autoimmune epilepsies already benefit from corticosteroids, IVIG, and monoclonal antibodies. Extending immune-targeted therapies to non-autoimmune epilepsy requires precision approaches.

Cutting-edge techniques—such as single-cell RNA sequencing, spatial proteomics, and iPSC-derived brain organoids—are beginning to map patient-specific immune–neuronal circuits and identify druggable targets (Danacikova et al., 2024). Integration of biomarker-guided stratification with temporal profiling of immune activation may enable tailored interventions that address both ictogenesis and disease progression.

Ultimately, the immune system is not peripheral to epilepsy—it is determinant of its onset, progression, and treatment responsiveness. Targeted immunomodulation, when combined with established ASDs, offers the potential to alter the natural history of TLE, reduce comorbidities, and achieve true disease modification. The challenge for the next decade is to translate mechanistic insights into therapies that reshape epileptogenesis rather than merely suppress its symptoms.

## 4 How inflammation shapes the development of epilepsy?

Epilepsy, particularly in its drug-resistant forms, is not solely a disorder of aberrant electrical activity but also one of dysregulated brain–immune interactions. Growing evidence implicates neuroinflammation—especially innate immune responses—as a driver of seizure initiation, progression, and chronicity. Inflammatory signalling, once regarded as secondary to seizures, is now recognized as a major determinant of epileptogenesis, particularly in acquired and lesional epilepsies characterized by BBB disruption and sustained glial activation. This conceptual shift arises from converging human, clinical, and experimental data, which identify TLRs, IL-1β, and the NLRP3 inflammasome as critical regulators of epileptogenesis (Vezzani et al., 2019; Vezzani et al., 2023; Danacikova et al., 2024; Wang et al., 2024).

### 4.1 Inflammation and epilepsy: lessons from human and animal studies

Histopathological analyses of resected brain tissue from patients with TLE consistently show active neuroinflammation, including microgliosis, astrogliosis, cytokine overexpression, and immune cell infiltration—even in the absence of infection or autoimmunity (Iori et al., 2016; Vezzani et al., 2023). Advanced imaging and biomarker studies link these inflammatory signatures to hippocampal atrophy, network reorganization, and cognitive dysfunction, underscoring their clinical relevance. Clinical studies further demonstrate that subsets of patients with refractory epilepsy respond to immunotherapies, suggesting that inflammation is not merely reactive but pathogenic (Vezzani et al., 2019). However, the incomplete and heterogeneous response to immunomodulation also indicates that inflammatory mechanisms are stratified across patients, reinforcing the need for biomarker-guided patient selection.

Preclinical models reinforce these observations. Chemoconvulsant-induced SE is associated with early glial activation, BBB breakdown, and marked cytokine release well before the onset of spontaneous seizures (Balosso et al., 2008; Noe et al., 2013). Targeted inhibition of inflammatory mediators—including TLR4 (Maroso et al., 2010), IL-1β (Balosso et al., 2008), and complement proteins—attenuates seizure severity and delays disease progression. These findings provide compelling evidence that inflammation is a causal factor in epileptogenesis, rather than an epiphenomenon. Viral encephalitis models and genetic susceptibility paradigms further show that distinct inflammatory programs can converge on similar epileptic phenotypes, highlighting neuroinflammation as a shared pathway across diverse aetiologies.

### 4.2 Cellular and molecular drivers of neuroinflammation

Microglia, the brain’s resident immune cells, detect neuronal stress through DAMPs via TLRs and NOD-like receptors. Their activation triggers the release of IL-1β, TNF-α, and IL-6, which heighten excitatory transmission and lower seizure thresholds (Vezzani et al., 2019). Astrocytes, when reactive, contribute further by releasing glutamate and reactive oxygen species, failing to regulate extracellular potassium, and amplifying cytokine-driven hyperexcitability (Bernardino et al., 2008; Noe et al., 2013). Together, glial responses remodel neural circuits, thereby perpetuating seizures. At the synaptic level, these cytokines modulate NMDA and AMPA receptor function, impair GABAergic inhibition, and alter the activity of voltage-gated sodium and potassium channels, thereby coupling inflammatory signals directly to changes in neuronal firing patterns and network oscillations. Importantly, glial cells can also assume anti-inflammatory or reparative states, characterized by IL-10 and TGF-β production and trophic support; in chronic TLE, these homeostatic programs appear blunted or dysregulated, favouring persistent pro-inflammatory signalling.

### 4.3 Blood–brain barrier dysfunction and complement activation

BBB dysfunction is a hallmark of epileptogenesis, permitting infiltration of peripheral immune cells and plasma proteins. These factors amplify local immune activation and sustain chronic inflammation. Complement component C3 is consistently elevated in both clinical and experimental epilepsy, facilitating endothelial dysfunction and leukocyte recruitment, thereby linking barrier failure with long-term neuroinflammation (Vezzani et al., 2019). Complement-mediated tagging and synapse elimination further contribute to aberrant circuit pruning, particularly in hippocampal and limbic networks. Imaging markers of BBB leakage and CSF–serum albumin ratios are emerging as candidate biomarkers to identify patients in whom barrier breakdown and complement activation are key drivers of disease progression.

### 4.4 Cytokines and chemokines as modulators of excitability

Cytokines act as potent modulators of network function. IL-1β, via IL-1R1, enhances glutamate release and promotes NMDA receptor phosphorylation, thereby driving excitatory overactivity (Balosso et al., 2008). TNF-α regulates AMPA receptor trafficking and promotes excitotoxicity, while IL-6 contributes to gliosis and cognitive decline (Kirkman et al., 2010). Chemokines, such as CXCL12 and CCL2, influence immune cell migration and also modulate synaptic plasticity and dendritic architecture, directly linking immune activation with circuit instability (Vezzani et al., 2019). These soluble mediators thus bridge systemic and central immune responses, translating peripheral inflammation into region-specific changes in excitability. Conversely, failure to appropriately terminate cytokine and chemokine production can fix pathological connectivity patterns, reinforcing seizure networks over time.

### 4.5 Toll-like receptors and the NLRP3 inflammasome: convergent pathways

TLRs serve as innate immune sentinels. TLR4, activated by HMGB1 released during seizures, induces NF-κB–dependent transcription of inflammatory mediators and perpetuates glial activation (Maroso et al., 2010). TLR9, which senses mitochondrial DNA, contributes to aberrant neurogenesis and persistent inflammation (Moresco et al., 2011). These pathways converge on the NLRP3 inflammasome, a cytosolic complex that activates caspase-1 to generate mature IL-1β and IL-18 (El-Sayed et al., 2023). Pharmacological inhibition of NLRP3 with MCC950 in pilocarpine-treated mice reduced seizure frequency, preserved cognition, and shifted microglia toward a reparative M2 phenotype—underscoring the inflammasome’s therapeutic potential (Wang et al., 2024). Emerging evidence suggests that endosomal nucleic acid–sensing receptors such as TLR3 and TLR7 may further couple viral or endogenous retroelement-derived RNA to inflammasome activation, although their precise roles in human TLE remain to be defined. Together, these data support a model in which distinct TLR pathways converge on shared effector nodes, providing multiple entry points for immune modulation but also emphasizing the need for pathway-specific targeting to avoid broad immunosuppression.

### 4.6 Targeting inflammation for disease modification

Several immune-based interventions show promise in modifying the course of epilepsy. Selective NLRP3 inhibition with MCC950 reduces both seizures and cognitive deficits in chronic TLE models (Wang et al., 2024). TLR4 blockade with TAK-242 attenuates HMGB1-driven excitability and preserves BBB integrity (Maroso et al., 2010). Early inhibition of IL-1R1 with agents such as anakinra or VX-765 decreases glial activation and neuronal death, although delayed treatment appears less effective (Noe et al., 2013). Complement inhibitors targeting C3a or C5a signalling prevent BBB breakdown and immune cell infiltration, while CSF1R inhibition reduces microglial proliferation and mitigates cognitive impairment (Vezzani et al., 2019). Crucially, these strategies aim to modulate rather than abolish immune function, preserving host defence and reparative mechanisms while dampening pro-epileptogenic signalling.

Technological advances now allow unprecedented resolution of neuroimmune dynamics. Single-cell transcriptomics and spatial proteomics have identified stage-specific glial phenotypes and immune cell signatures that correlate with seizure severity (Kumar et al., 2022; Hanin et al., 2024). Patient-derived iPSC-based brain organoids and humanized mouse models further enable mechanistic dissection of patient-specific immune–neuronal interactions, accelerating biomarker-driven drug discovery (Wu et al., 2025). In parallel, candidate biomarkers—including CSF and serum cytokine profiles, HMGB1 and complement fragments, TSPO-PET imaging of microglial activation, and immune-coupled EEG signatures—are beginning to define measurable endpoints for early-phase trials of immunomodulatory therapies.

### 4.7 Towards a new therapeutic paradigm

Inflammation is not a by-product of epilepsy but a central mechanism in its initiation and progression. By altering cytokine balance, glial function, barrier integrity, and receptor signalling, neuroinflammation reshapes neuronal circuits to favour hyperexcitability and chronicity (Dingledine et al., 2024). Human and experimental studies converge on immune pathways—particularly IL-1β, TLRs, and the NLRP3 inflammasome—as tractable therapeutic targets (Chen et al., 2024). Emerging work on the gut–brain axis further suggests that microbial metabolites and diet can tune central immune tone and seizure susceptibility, opening additional avenues for intervention.

The next decade should prioritize translating these mechanistic insights into biomarker-guided immunotherapies that can be integrated with antiseizure drugs. Such therapies will likely require precise timing, patient stratification based on immune signatures, and CNS-selective delivery platforms. If successful, this strategy offers the prospect of true disease modification, attenuation of cognitive and psychiatric comorbidities, and a fundamental shift in the management of drug-resistant epilepsy—from symptomatic control to targeted immunological intervention.

## 5 Neurogenesis and immune system interactions

**FIGURE 2:**
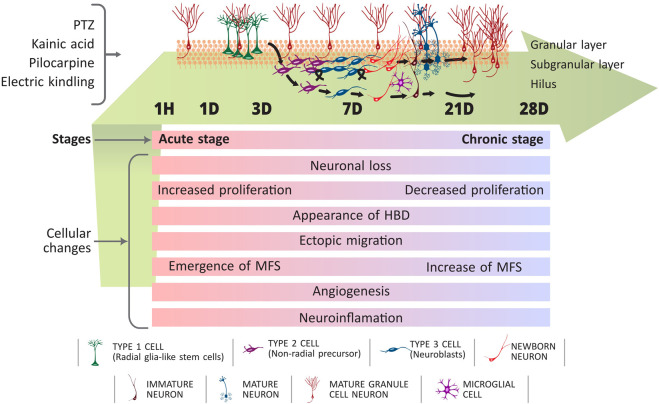
Spatiotemporal disruption of hippocampal neurogenesis and maladaptive circuit remodeling during epileptogenesis. Epileptogenic insults—such as PTZ, kainate, pilocarpine, or electrical kindling—trigger rapid alterations in the subgranular and granular layers of the dentate gyrus. Within hours to days, inflammatory mediators (e.g., IL-1β, TNF-α, ROS) and excitotoxic signalling increase proliferation of radial glia-like type-1 cells and non-radial type-2 progenitors, leading to excess generation of neuroblasts (type-3 cells). During the early latent phase (1H–7D), newborn dentate granule cells (DGCs) exhibit hallmarks of pathological maturation, including ectopic migration into the hilus and formation of aberrant basal dendrites (HBD). By the subacute phase (7–21D), these displaced and immature neurons integrate abnormally into hippocampal circuits, contributing to the emergence of mossy fibre sprouting (MFS) and establishing recurrent excitatory pathways. Microglial activation, angiogenesis, and neuroinflammation further distort the neurogenic niche. In the chronic stage (21–28D), NPC proliferation declines, yet maladaptive circuitry persists, with immature and mature DGCs aberrantly reinforcing hyperexcitable networks. Progressive expansion of MFS and sustained glial activity contribute to long-term hippocampal instability. Together, these structural and cellular changes illustrate how epileptogenesis redirects neurogenesis from an adaptive, plasticity-supporting process toward maladaptive rewiring. Modulating neuroinflammation—via IL-1R1 inhibition, TLR pathway blockade, or microglial phenotypic reprogramming—may restore healthy integration of newborn neurons and reduce seizure susceptibility.

**FIGURE 3:**
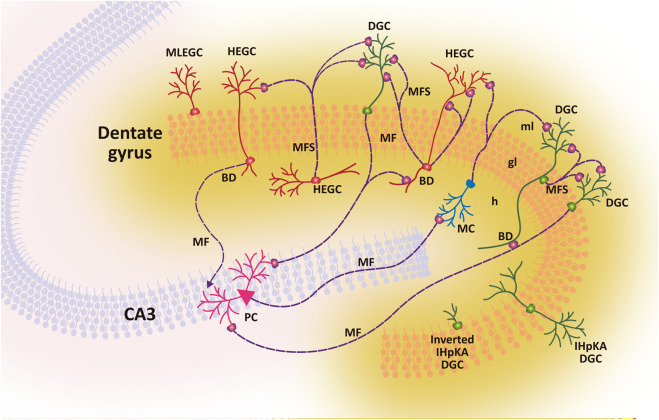
Maladaptive circuit remodeling underlying hippocampal hyperexcitability in temporal lobe epilepsy. Epileptogenic insults induce profound structural reorganization within the dentate gyrus and CA3 circuitry. Persistent activation of innate immune pathways—including IL-1R1, TLR4, and NLRP3—alters the maturation and positioning of dentate granule cells (DGCs), resulting in ectopic migration into the hilus and molecular layer, the generation of hilar ectopic granule cells (HEGCs), and the appearance of molecular layer ectopic granule cells (MLEGCs). Newborn and mature DGCs frequently develop aberrant basal dendrites that receive inappropriate excitatory input, while mossy fibre sprouting (MFS) forms recurrent excitatory loops among HEGCs, mossy cells (MCs), and CA3 pyramidal neurons (PCs). These rewired circuits bypass the dentate gate, enabling direct excitation of CA3 via aberrant mossy fibre projections and multi-synaptic feedback loops involving HEGCs and inverted IHpKA-induced DGCs. Microglial activation and neuroinflammatory signalling reinforce this instability by promoting synaptic reorganization and dendritic remodeling. The cumulative effect is a self-perpetuating excitatory network that lowers seizure threshold, drives spontaneous recurrent seizures, and contributes to cognitive decline. Together, these alterations illustrate how chronic neuroinflammation and innate immune signalling reshape hippocampal architecture, shifting the dentate gyrus from a gatekeeper of excitability to a generator of recurrent activity. Therapeutic targeting of glial–neuronal inflammatory crosstalk may prevent or reverse maladaptive circuit formation and protect hippocampal function.

Neurogenesis and innate immune signalling intersect to shape hippocampal plasticity in epilepsy, converting a normally reparative programme into one that favours maladaptive circuit remodelling. In the healthy adult brain, neurogenesis is primarily concentrated in the dentate gyrus subgranular zone and the subventricular zone, where metabolic, environmental, and immune cues collectively sustain cognitive flexibility (Figure 2). In TLE, seizure-associated inflammation disrupts these niches, promoting ectopic migration, abnormal dendritic architecture, and faulty synaptic incorporation of newborn neurons (Figure 3)—changes associated with hyperexcitability and cognitive decline (Chen et al., 2021; Chen et al., 2024; Dingledine et al., 2024). These alterations highlight how inflammatory mediators can transform an intrinsically adaptive regeneration programme into a source of long-lasting circuit instability.

### 5.1 From repair to network instability

Longitudinal imaging and single-cell analyses reveal a stage-dependent trajectory: an early, seemingly compensatory rise in neurogenesis after SE is followed by dentate granule cell misplacement, mossy fibre sprouting, and disordered dendritic remodelling that destabilise hippocampal circuits and persist into chronic epilepsy (Chen et al., 2021). Although enhanced neurogenesis can support recovery in select contexts, most evidence indicates that aberrant integration of immature neurons sustains epileptogenesis, reflecting how the inflammatory milieu skews the balance between adaptive repair and pathological plasticity (Vezzani et al., 2019; Chen et al., 2024). Notably, the timing of neurogenic bursts relative to inflammatory cascades appears critical: early neurogenesis in a permissive environment may be reparative (Figure 2), whereas delayed neurogenesis in a cytokine-rich milieu promotes faulty integration and enduring hyperexcitability (Figure 3).

### 5.2 Innate immune control of the neurogenic niche

Microglia are principal gatekeepers of the neurogenic microenvironment. Seizure-driven microglial activation elevates the production of IL-1β, TNF-α, and IL-6, thereby suppressing neural progenitor proliferation and differentiation. In contrast, in alternative activation states, microglia secrete trophic factors that can support neurogenesis, underscoring a context-dependent bidirectionality (Deng et al., 2020; Broer and Pauletti, 2024). Astrocytes amplify inflammatory tone—releasing glutamate, reactive oxygen species, and cytokines—and exhibit distinct disease-associated states on single-cell profiling that differentially influence progenitor dynamics and synaptic function (Habib et al., 2020; Piwecka et al., 2023). Disruption of glial homeostatic programs, including impaired IL-10 and TGF-β signalling, further reduces the brain’s capacity to stabilize newly generated neurons, biasing the niche toward maladaptive growth.

### 5.3 Toll-like receptors: immune gatekeepers of plasticity

TLRs link innate immunity to neurogenic control. TLR4, activated by HMGB1 released during seizures, drives NF-κB–dependent transcription that impairs progenitor proliferation and biases toward aberrant rewiring; pharmacological TLR4 blockade (e.g., resatorvid/TAK-242) reduces seizures and improves cognition in preclinical models (Maroso et al., 2010; Dong et al., 2022). TLR3 signalling induces type I interferons, which suppress neurogenesis and worsen memory performance (Okun et al., 2010). TLR9 exerts nuanced effects—its activation can both raise TNF-α and restrain seizure-induced aberrant neurogenesis by limiting progenitor proliferation, suggesting context-specific neuroprotection (Moresco et al., 2011). TLR2, enriched in activated microglia in epileptic hippocampus, contributes to neuronal injury; TLR2 inhibition decreases gliosis and seizure burden in models (Babcock et al., 2006). Collectively, these pathways illustrate that TLRs act as precision regulators of neurogenic output, translating inflammatory signals into enduring structural changes that shape excitability. Moreover, recent evidence implicates endosomal TLRs—particularly TLR7—in responding to endogenous retroelement-derived RNA, suggesting that nucleic acid–sensing pathways link genomic stress to maladaptive neurogenesis in TLE.

### 5.4 Inflammasome signalling and disease modification

TLR inputs converge on the NLRP3 inflammasome, which catalyses caspase–1–dependent IL-1β maturation (El-Sayed et al., 2023). Selective NLRP3 inhibition with MCC950 in pilocarpine-treated mice reduces seizure frequency, lowers hippocampal IL-1β, shifts microglia toward reparative states, and preserves cognition—supporting inflammasome signalling as a tractable, disease-modifying axis (Haque et al., 2024; Wang et al., 2024). Given NLRP3’s central role as an integrator of metabolic stress, ionic imbalance, and DAMP signalling, inflammasome inhibition may simultaneously stabilise neurogenic niches and reduce excitability, offering a dual-acting therapeutic approach.

### 5.5 Translational avenues: preserving adaptive neurogenesis while curbing inflammation

Therapeutic strategies now target neuroimmune checkpoints that promote neurogenesis toward repair. For instance, IL-1R1 blockade can rescue progenitor function and synaptic integrity (Smirnova and Quan, 2025). Additionally, CSF1R inhibitors modulate microglial populations and phenotype, albeit with important caveats regarding region specificity and off-target myeloid effects (Geng et al., 2019). TLR4 antagonists (TAK-242) dampen HMGB1-driven hyperexcitability and protect the BBB (Maroso et al., 2010; Dong et al., 2022). Minocycline, a microglial modulator, limits aberrant neurogenesis and chronic inflammation in seizure models and is under clinical exploration for inflammatory epilepsies (Victor and Tsirka, 2020). Emerging metabolic approaches—including ketogenic interventions and gut–brain modulation—may also normalize neurogenic dynamics by altering systemic cytokine tone. Delivery innovations, including nanoparticle-mediated CNS targeting and engineered biologics with enhanced brain penetrance, are poised to accelerate translation while minimizing systemic immunosuppression (Lee et al., 2023; Piwecka et al., 2023).

### 5.6 Tools that enable precision neuroimmunology

Single-cell and spatial transcriptomics now resolve the cell-state trajectories of microglia, astrocytes, and progenitors across epileptogenesis, identifying inflammatory signatures that correlate with seizure severity (Kumar et al., 2022; Piwecka et al., 2023). Patient-derived brain organoids and humanized mouse models capture patient-specific neuroimmune interactions, supporting biomarker-guided drug discovery. Multiplex cytokine profiling, TSPO-PET imaging of microglial activation, and circulating microRNA signatures are emerging as complementary biomarkers that can identify individuals in whom neuroinflammatory forces dominate epileptogenic progression. Computational models that integrate cytokine signalling, glial state transitions, and neurogenic flux are emerging to predict response and guide trial design (Vezzani et al., 2019). Future platforms that couple computational prediction with real-time neurophysiological monitoring may allow adaptive, personalised immunomodulation in clinical practice.

### 5.7 Toward a new paradigm

Neurogenesis in epilepsy is a decisive balance between repair and pathology, governed by cytokine gradients, microglial reactivity, and TLR-inflammasome signalling (Al-Dhahi et al., 2025). By pairing antiseizure therapies with biomarker-guided immunomodulation that preserves adaptive neurogenesis and prevents maladaptive integration, clinicians can move beyond symptomatic control toward disease modification. This integrated strategy reframes TLE as a disorder of immune–circuit dysregulation, in which correcting inflammatory tone is essential to stabilizing plasticity and restoring cognitive function. Ultimately, preserving the adaptive facets of neurogenesis while constraining inflammation-driven maladaptation defines the next Frontier in precision neurology for drug-resistant epilepsy.

## 6 Inflammatory molecules in chronic epilepsy

Neuroinflammation is increasingly recognized as a central determinant in the evolution of epilepsy, driving the transition from an acute insult to a chronic, treatment-resistant disorder. Key innate immune sensors (notably TLR4 and IL-1R1) detect endogenous danger signals and trigger inflammatory cascades that generate pro-inflammatory cytokines, interferons, and chemokines. This cascade sustains microglial and astrocytic activation, disrupts BBB integrity, and enhances neuronal hyperexcitability. Rather than representing a by-product of seizures, these processes form a self-perpetuating feedback loop that fuels recurrent seizures and the chronic neuroinflammatory milieu characteristic of drug-resistant epilepsy (Vezzani et al., 2019; Dong et al., 2022; Wang et al., 2024). This chronic inflammatory state also correlates with cognitive impairment and mood disturbances, underscoring the systemic neurological consequences of persistent glial activation.

### 6.1 Cytokine signalling and neurotransmitter dysregulation

Among pro-inflammatory mediators, IL-1β has emerged as a critical regulator of excitability. Preclinical work shows that IL-1β potentiates NMDA receptor–mediated currents in hippocampal CA1 neurons and reduces outward potassium conductance, thereby amplifying hyperexcitability (Trevino et al., 2007). In parallel, IL-1β impairs GABAergic inhibition through TLR4-dependent mechanisms, further disrupting excitatory–inhibitory balance (Balosso et al., 2008; Vezzani et al., 2019). These combined effects lower the seizure threshold and facilitate the propagation of seizures across hippocampal and cortical circuits. IL-1β additionally influences neurovascular coupling and metabolic support, further destabilising neuronal homeostasis.

Therapeutic targeting of IL-1R1 has produced mixed results. IL-1 receptor antagonists (anakinra, VX-765) confer neuroprotection when administered before or shortly after SE but show limited efficacy in established epilepsy, with little effect on spontaneous recurrent seizures (Noe et al., 2013). This discrepancy underscores the importance of therapeutic timing: early blockade may prevent maladaptive network reorganization, whereas delayed intervention appears insufficient to reverse entrenched pathology (Haque et al., 2024). The time-sensitive nature of IL-1β signalling thus suggests a critical therapeutic window early in epileptogenesis during which cytokine modulation may meaningfully influence disease trajectory.

### 6.2 Microglial activation and TNF-α signalling

Chronic microglial activation sustains inflammation by releasing TNF-α, IL-6, and reactive oxygen species (ROS). TNF-α, acting via TNFR1, alters AMPA receptor trafficking, enhances synaptic plasticity, and increases excitatory drive, thereby contributing to both acute seizure susceptibility and long-term instability (Terreros-Roncal et al., 2021). However, TNF-α is not exclusively deleterious. Experimental inhibition of TNF-α signalling in chronic epilepsy models sometimes exacerbates aberrant neurogenesis, leading to ectopic neuronal migration and maladaptive excitatory connectivity (Broer and Pauletti, 2024). This dual role highlights the stage-specific and cell-type-dependent actions of TNF-α, suggesting that indiscriminate inhibition may be counterproductive. A refined understanding of TNF-α′s bifunctional roles—pro-epileptogenic vs. neuroprotective—will be crucial for the development of selective modulators that target pathological signalling while preserving compensatory mechanisms.

### 6.3 TLR4 signalling and the transition to chronic epilepsy

Accumulating evidence indicates that TLR4 is central to the epileptogenic cascade. By recognizing damage-associated molecular patterns such as HMGB1, TLR4 activates NF-κB signalling, upregulates IL-1β and TNF-α, disrupts BBB integrity, and exacerbates astrocytic reactivity and glutamate dysregulation (Maroso et al., 2010; Dong et al., 2022). These effects promote excitotoxicity and maladaptive remodelling of hippocampal networks, facilitating the shift from acute to chronic epilepsy. Pharmacological inhibition of TLR4 with selective antagonists such as TAK-242 reduces seizure frequency, preserves BBB function, and mitigates neuroinflammation in rodent models (Maroso et al., 2010; Dong et al., 2022). TLR4 also regulates microglial phagocytic behaviour and complement activation, positioning it as a master controller of neuroimmune–synaptic interactions. Disrupting the HMGB1–TLR4 axis thus represents a compelling therapeutic strategy, particularly when combined with agents that reinforce BBB stability to limit peripheral immune infiltration and downstream network injury.

### 6.4 Combinatorial strategies: beyond monotherapy

Despite compelling mechanistic evidence, anti-inflammatory monotherapies have shown limited benefit in established epilepsy. However, combining immune-targeting agents with ASMs or neuroprotective compounds represents a rational approach. For instance: a) IL-1β antagonism combined with AMPA receptor modulators may restore synaptic balance. b) TNF-α inhibition coupled with neurotrophic support could counter excitotoxicity while preserving adaptive plasticity. c) TLR4 blockade integrated with BBB stabilizers may prevent chronic immune infiltration and reduce long-term seizure burden. d) NLRP3 inflammasome inhibition combined with metabolic modulators may reduce both excitability and inflammation by targeting convergent stress pathways (El-Sayed et al., 2023).

Such combinatorial strategies directly address both symptomatic seizures and the underlying disease mechanisms, offering greater potential for disease modification (Vezzani et al., 2019; Haque et al., 2024). Future regimens may incorporate sequence-specific timing, targeting distinct inflammatory nodes at defined stages of epileptogenesis to maximise therapeutic effect.

### 6.5 Future directions: biomarkers and precision immunotherapy

To move beyond empiricism, biomarker-driven stratification is essential. Identifying patients based on inflammatory signatures—whether through single-cell transcriptomics, spatial proteomics, or advanced neuroimaging—will enable clinicians to tailor therapies to the disease stage and immune profile (Kumar et al., 2022; Piwecka et al., 2023). Biomarkers of glial reactivity or cytokine dysregulation could inform early intervention strategies, improving the likelihood of preventing epileptogenesis. Circulating cytokine panels, CSF inflammatory markers, complement fragments, extracellular HMGB1, and TSPO-PET imaging are among the most promising candidates for stratifying patients according to dominant inflammatory pathways.

Innovations in nanoparticle-based drug delivery are improving therapeutic specificity, reducing systemic immunosuppression, and enhancing CNS penetration (Veloz-Castillo et al., 2016; Li et al., 2024). These platforms enable selective modulation of activated microglia and reactive astrocytes while preserving essential immune functions. Patient-derived organoids and humanised mouse models provide additional translationally relevant systems for preclinical evaluation (Wu et al., 2025). Integration of computational modelling with real-time biomarker monitoring could permit adaptive immunotherapy, dynamically matching treatment intensity to the inflammatory trajectory.

Chronic neuroinflammation in epilepsy is not a secondary phenomenon but a driver of disease progression, network instability, and cognitive decline. Key mediators—including IL-1β, TNF-α, and TLR4—offer tractable therapeutic targets, but efficacy hinges on timing, cellular specificity, and combination with standard ASMs. As biomarker discovery and precision delivery technologies advance, immunotherapy could shift the clinical paradigm from symptomatic seizure suppression toward disease modification, cognitive preservation, and long-term remission.

## 7 Redefining epilepsy through immune modulation

Epilepsy is increasingly recognized not only as a disorder of aberrant electrical discharges but also as a disease of chronic immune dysregulation. Evidence shows that seizures perpetuate, and are perpetuated by, sustained neuroinflammation, creating a vicious cycle that drives progression and pharmacoresistance (Dingledine et al., 2024). This recognition reframes epilepsy as a disorder of both excitability and immunity, placing the immune system at the centre of therapeutic innovation. By disrupting maladaptive inflammatory loops, restoring BBB integrity, rebalancing cytokine signaling, and recalibrating glial–neuronal interactions, immune-targeted therapies have the potential not only to suppress seizures but also to interrupt epileptogenesis and radically modify disease trajectory. This dual perspective—addressing electrical instability and immune dysfunction—marks a conceptual pivot toward a systems-level understanding of epilepsy.

### 7.1 Precision modulation of Toll-like receptors

TLRs have emerged as upstream orchestrators of seizure-induced inflammation. TLR4, activated by HMGB1 and other DAMPs, triggers NF-κB signalling, induces cytokine release, disrupts glutamate homeostasis, and compromises BBB integrity. Pharmacological blockade with TAK-242 (resatorvid) reduces IL-1β and TNF-α release, preserves synaptic integrity, and restores BBB function, producing seizure protection in rodent models (Dong et al., 2022; Li et al., 2023).

Other TLRs also regulate epileptogenesis. TLR2, upregulated in activated microglia, promotes hippocampal inflammation; its inhibition dampens gliosis and seizure burden (Medel-Matus et al., 2017). TLR9, which senses mitochondrial DNA, exerts context-specific effects—limiting seizure-induced aberrant neurogenesis in some models while promoting neurotoxicity in others (Moresco et al., 2011; Piwecka et al., 2023). TLR3 and TLR7, both endosomal nucleic acid sensors, may integrate viral mimics and endogenous retroelement activity into inflammatory cascades, suggesting unexplored intersections between antiviral immunity and epileptogenesis. The challenge ahead is to decode patient-specific TLR activity, identify when TLR signalling is pathological, compensatory, or protective, and use this information to target maladaptive rewiring while precisely preserving essential immune surveillance.

### 7.2 Cytokine neutralization: strategic timing matters

Pro-inflammatory cytokines amplify seizure susceptibility through diverse mechanisms. IL-1β enhances NMDA receptor currents, reduces potassium conductance, and impairs GABAergic inhibition, thereby tipping the excitatory–inhibitory balance (Balosso et al., 2008; Vezzani et al., 2019). IL-1 receptor antagonists (IL-1Ra, such as anakinra) reduce neuronal death and inflammation when administered early after SE, but show limited efficacy in established epilepsy, underscoring the critical importance of timing (Noe et al., 2013; Haque et al., 2024). TNF-α signalling via TNFR1 alters AMPA receptor trafficking, increases excitability, and drives gliosis (Terreros-Roncal et al., 2021). Nevertheless, TNF-α also regulates neurogenesis, and indiscriminate inhibition may worsen maladaptive plasticity (Broer and Pauletti, 2024). Similarly, IL-6 promotes BBB disruption and cognitive impairment, but also mediates repair under certain contexts (Che et al., 2024).

These dual and context-specific actions highlight that cytokines cannot be classified as “beneficial” or “harmful.” Effective cytokine modulation requires stage-specific, biomarker-guided algorithms that target pathological signalling without suppressing essential reparative or homeostatic roles. This paradigm underscores the importance of timing, dosage, and patient stratification in cytokine-based intervention.

### 7.3 Broad-spectrum immunomodulators: restoring balance

Several repurposed agents exert broad immunomodulatory effects. Minocycline reduces microglial activation, IL-6 release, and ROS production, attenuating aberrant neurogenesis and improving cognitive outcomes in chronic models (Victor and Tsirka, 2020). Dexamethasone limits cytokine release and leukocyte infiltration, providing short-term neuroprotection but with systemic liabilities. IFN-β strengthens BBB integrity, modulates cytokine production, and reduces seizure burden while preserving immune defence, positioning it as a dual-action candidate in drug-resistant epilepsy (Han et al., 2024). These agents illustrate the value of rebalancing—rather than globally suppressing—immune activity. Their mechanisms engage multiple inflammatory nodes simultaneously, offering broader benefits in patients with diffuse, chronic, or poorly defined neuroinflammation. The challenge is to retain this broad utility while minimizing systemic effects through targeted delivery or CNS-selective formulations.

### 7.4 Combination therapies: synergy over monotherapy

Monotherapies targeting inflammation have shown modest efficacy once chronic epilepsy is established. A more effective approach may lie in synergistic combinations that simultaneously address excitability and inflammation: a) IL-1Ra + ASMs: enhances seizure control while reducing neurodegeneration (Noe et al., 2013). b) TNF-α inhibitors + neurotrophic agents: mitigate excitotoxicity while preserving plasticity. c) TLR4 antagonists + BBB stabilizers: prevent immune infiltration and sustain CNS homeostasis (Dong et al., 2022). d) NLRP3 inhibitors + metabolic modulators: dampen excitability and reduce inflammasome activity through complementary pathways.

Such strategies aim not only to treat seizures but also to shift disease trajectory, delaying or preventing chronic epilepsy in high-risk individuals. This combination-based logic mirrors the logic of immuno-oncology approaches, in which targeting multiple pathways simultaneously produces durable disease control.

### 7.5 Toward personalized immunotherapy

Immune responses vary across patients, influenced by genetic background, comorbidities, and epigenetic states. To move beyond one-size-fits-all interventions, therapies must be guided by immune phenotyping. Advances in single-cell transcriptomics, spatial proteomics, and longitudinal neuroimaging are defining inflammatory states across epilepsy stages and linking them to seizure severity (Kumar et al., 2022; Piwecka et al., 2023). Patient-derived organoids and humanized mouse models now enable validation of precision interventions in genetically relevant contexts (Wang et al., 2025).

Nanoparticle-based delivery platforms are enhancing CNS penetration and cell-type specificity, enabling local modulation of activated microglia or astrocytes while sparing systemic immunity (Veloz-Castillo et al., 2016). CRISPR-based epigenetic editing, engineered biologics with enhanced brain penetrance, and peripheral-to-CNS signalling inhibitors are emerging as next-generation immunological tools with high translational promise. In parallel, AI-driven modelling of immune–neural dynamics promises to guide real-time treatment choices, predict disease course, and stratify patients based on likely therapeutic response.

Epilepsy can no longer be viewed solely through the lens of hyperexcitability. It is also a disease of chronic neuroinflammation, in which TLR signalling, cytokine cascades, complement activity, and glial dysfunction perpetuate progression (Shi et al., 2025). Targeting these mechanisms with precision immunotherapies—tailored by biomarkers, delivered with nanotechnology, and combined with ASMs—holds the potential to interrupt epileptogenesis, preserve cognition, and achieve durable remission.

Immune-targeting strategies are not peripheral; they are central to the next therapeutic revolution in epilepsy (Ravikumar et al., 2025). The future of care lies in integrating immunological insight with technological innovation, shifting the paradigm from symptomatic seizure suppression to disease modification and restoration of neural homeostasis.

## 8 The TLR7/endogenous retrovirus axis: toward a new immunogenomic framework in epileptogenesis

A growing body of evidence suggests that nucleic acid–sensing pathways may play a previously underappreciated role in epileptogenesis, linking viral immunity, endogenous retroelement biology, and chronic neuroinflammation (Romer, 2021). Among these pathways, TLR7—a sensor of single-stranded RNA primarily expressed in microglia, astrocytes, and infiltrating myeloid cells—has received comparatively little attention in epilepsy research—nevertheless, emerging mechanistic insights position TLR7 as a potential integrator of genomic stress and innate immune activation (Gantier et al., 2008; Yu et al., 2012; Vargas-Calderon et al., 2024). Here, we hypothesize that dysregulated interactions between TLR7 and ERVs—genomic remnants of ancient viral integrations—may constitute a previously unrecognised axis contributing to TLE-related seizure susceptibility, persistent inflammation, and maladaptive circuit remodelling.

ERVs represent approximately 8% of the human genome and are typically silenced through DNA methylation, chromatin compaction, and RNA degradation pathways such as those mediated by Regnase-1/Roquin endonucleases (Yu et al., 2012; Yoshinaga and Takeuchi, 2024). Under conditions of physiological stress, infection, oxidative injury, or cytokine-mediated chromatin relaxation, these normally quiescent sequences may become transcriptionally active (Yu et al., 2012). Reactivation generates single-stranded RNA species with GU-rich motifs structurally similar to viral genomes—precisely the ligands recognized by TLR7 (Liu et al., 2016). In microglia, TLR7 engagement triggers MyD88-dependent activation of NF-κB and IRF7 pathways, promoting the release of IL-6, type I interferons (IFN-α/β), and downstream inflammasome components. These mediators alter synaptic homeostasis, enhance glutamate release, disrupt GABAergic inhibition, and bias neural networks toward hyperexcitability, thereby recapitulating molecular signatures characteristic of early epileptogenesis (Vezzani et al., 2019; Wang et al., 2024).

Observations in other neuroinflammatory disorders further support the potential link between TLR7 signalling and ERV activity. In systemic lupus erythematosus, TLR7 overactivation drives aberrant IFN-1 production and is associated with cognitive impairment and cortical excitability (Zhang and Casanova, 2024). In neurodegenerative models, including frontotemporal dementia, ERV transcripts accumulate in microglia and induce TLR7-dependent inflammatory signatures similar to those observed in chronic epilepsy (Hsieh et al., 2020). Viral encephalitis models provide additional insight: Theiler’s murine encephalomyelitis virus infection alters TLR7 responses and leads to persistent limbic seizures, suggesting that nucleic acid–sensing receptors shape long-term excitability following infection (Kirkman et al., 2010). These convergent lines of evidence imply that TLR7 may serve as a general amplifier of chronic neuroinflammation when exposed to persistent or inadequately regulated RNA stimuli.

In this framework, TLR7 dysregulation could promote epileptogenesis through at least two complementary mechanisms. First, hyperactive TLR7 signalling may exaggerate microglial cytokine and interferon responses, sustaining an inflammatory milieu that lowers seizure threshold, disrupts synaptic plasticity, and destabilizes hippocampal networks. Second, impaired TLR7 function—or deficiencies in associated RNA degradation pathways—may permit intracellular accumulation of ERV-derived RNA, which can activate inflammasomes such as NLRP3 or the cGAS–STING pathway, thereby driving sustained inflammation even in the absence of exogenous pathogens. Such a model aligns with recent evidence showing NLRP3 upregulation in human TLE hippocampi and robust anti-convulsant effects of inflammasome inhibitors such as MCC950 (Cristina de Brito Toscano et al., 2021; Wang et al., 2024).

If the TLR7/ERV axis contributes to epileptogenesis, several experimentally testable predictions emerge. Transcriptomic profiling of resected TLE tissue should reveal elevated ERV RNA signatures in microglia and astrocytes, accompanied by enrichment for markers of TLR7–MyD88–IRF7 pathway activation. Genetic studies may identify TLR7 polymorphisms or mutations in RNA regulatory genes (e.g., TREX1, ADAR1, Regnase-1) that lower RNA tolerance thresholds, predisposing certain individuals to chronic epilepsy after an acute insult. *In vivo*, TLR7-deficient mice exposed to chemoconvulsants or viral triggers should display altered cytokine landscapes and distinct seizure phenotypes compared with wild-type controls, whereas pharmacological TLR7 antagonism may attenuate microglial reactivity, reduce IFN-I signalling, and mitigate network hyperexcitability.

Should this model be validated, the therapeutic implications would be profound. Targeting nucleic acid–sensing pathways would move epilepsy treatment beyond classical cytokine blockade toward modulation of upstream immunogenomic triggers. Small-molecule TLR7 antagonists, already in development for autoimmune disease, could dampen inappropriate recognition of endogenous RNA. Reverse transcriptase inhibitors—effective in suppressing ERV activity in neuroinflammatory and neurodegenerative contexts—could prevent ERV-driven TLR7 activation. Epigenetic therapies that restore ERV silencing, or RNA-targeting approaches such as antisense oligonucleotides, may further suppress pathogenic RNA accumulation. Crucially, these strategies would aim not to broadly suppress immunity but to normalize aberrant intracellular sensing of self-derived nucleic acids, thereby re-establishing immunological tolerance within neural circuits.

In summary, the TLR7/ERV axis provides a unifying conceptual framework linking genomic instability, innate immune activation, and hippocampal hyperexcitability. Although speculative, this model integrates well-established immunological mechanisms with emerging observations in epilepsy, autoimmunity, and neurodegeneration. By reframing epileptogenesis as a disorder of dysregulated RNA sensing, it opens novel avenues for biomarker discovery, patient stratification, and therapeutics. Future studies applying single-cell transcriptomics, spatial proteomics, and organoid-based modelling will be essential to determine whether TLR7–ERV interactions represent a core pathogenic axis or a disease-modifying pathway in a subset of patients with drug-resistant TLE.

## 9 Discussion

Neuroinflammation is now recognised as a primary driver of epileptogenesis and progression rather than a secondary consequence of seizures (Shi et al., 2025). In TLE, innate immune signalling—particularly through TLRs, proinflammatory cytokines, and complement activation—establishes a self-reinforcing inflammatory microenvironment that promotes recurrent seizures, accelerates synaptic reorganisation, and fosters pharmacoresistance (Vezzani et al., 2019; Vezzani et al., 2023; Fawzy et al., 2025; Ravikumar et al., 2025). This conceptual shift reframes TLE as a disorder of both excitability and chronic immune dysregulation, with mechanistic and therapeutic implications that extend beyond classical electrophysiological models.

Conventional chemoconvulsant paradigms (pilocarpine, kainate) have been fundamental in establishing immune mechanisms underlying epileptogenesis, revealing stereotyped cascades of microgliosis, astrogliosis, BBB disruption, and cytokine elevation. However, their abrupt, widespread neuronal injury does not fully recapitulate the temporally extended, heterogeneous progression of human TLE, nor do they capture genetic susceptibility, systemic immune contributors, or environmental modulators (Levesque et al., 2016; Loscher and Howe, 2022). Newer platforms—including patient-specific iPSC-derived organoids (Wu et al., 2025) and humanised immune-competent mouse models (Terreros-Roncal et al., 2021)—enable more precise dissection of cell-type-specific neuroimmune interactions and genotype–phenotype relationships, though scalability and reproducibility remain developmental challenges.

Neuroinflammatory responses vary significantly across disease stages. Early, acute activation of IL-1β, TNF-α, and IL-6 contributes to an excitatory–inhibitory imbalance through NR2B phosphorylation, changes in AMPA receptor trafficking, modulation of potassium channels, and impaired GABAergic transmission (Balosso et al., 2008; Terreros-Roncal et al., 2021; Piwecka et al., 2023). As disease progresses, glial phenotypes diversify: microglia may polarise toward M1-like inflammatory states or adopt reparative M2-associated signatures, while astrocytes exhibit subtype-specific responses that either exacerbate or constrain network instability (Deng et al., 2020; Broer and Pauletti, 2024). Spatial transcriptomics has revealed that these states are highly compartmentalised within hippocampal subregions and evolve dynamically (Kumar et al., 2022), underscoring the need for temporally calibrated intervention.

The therapeutic efficacy of immune modulation is profoundly time-dependent. IL-1R1 antagonists and caspase-1 inhibitors attenuate neuronal loss and reduce acute seizure severity when administered during or shortly after SE but show inconsistent effects once spontaneous recurrent seizures are established (Noe et al., 2013; Haque et al., 2024; Wang et al., 2024). Similarly, NLRP3 inflammasome inhibition effectively dampens early neuroinflammation and preserves cognition but appears less impactful once chronic microglial activation becomes autonomous (El-Sayed et al., 2023). Biomarker-informed timing will therefore be essential. Advanced PET ligands targeting TSPO, IL-1β, TLR4, and NLRP3 (Brackhan et al., 2016) offer a route to define therapeutic windows and track treatment responses with unprecedented precision.

Even with validated molecular targets, drug delivery poses a fundamental obstacle. TLR4 antagonists and HMGB1-neutralising agents show potent anti-inflammatory and anti-seizure effects in rodents, but face limited BBB penetration and systemic toxicity in humans (Dong et al., 2022). Nanoparticle-based strategies—including liposomal, polymeric, and exosome-derived carriers—have begun to overcome these pharmacokinetic limitations by enabling targeted delivery to microglia, astrocytes, or endothelium (Piwecka et al., 2023). As these technologies mature, cell-type specificity and controlled release may allow for circuit-selective modulation without compromising systemic immunity.

Systemic immune networks also contribute to seizure susceptibility. Dysbiosis alters the availability of short-chain fatty acids (SCFAs), influencing microglial tone and shaping TLR responsiveness in both peripheral and central immune cells (Ding et al., 2021). Experimental correction of dysbiosis through probiotics or faecal microbiota transplantation reduces seizure burden in rodent models, suggesting that gut-derived metabolic cues may modulate neuroinflammatory thresholds. How these peripheral signals interact with CNS-intrinsic immune pathways represents a key Frontier.

An underexplored yet potentially transformative mechanism involves TLR7 in detecting single-stranded RNA (ssRNA). Although originally characterised as an antiviral receptor, TLR7 also participates in immunosurveillance of ERVs. In B cells, TLR7-dependent recognition of ERV-derived antigens promotes antibody-mediated containment of ERV reactivation, thereby preventing aberrant immune activation (Crozat and Beutler, 2004; Yu et al., 2012). Human data show that rare TLR7 loss-of-function variants predispose to severe viral infection by impairing type-I interferon induction (Mantovani et al., 2022; Antoli et al., 2025), suggesting that TLR7 is crucial for maintaining the balance between antiviral and autoreactive immunity.

Here, we propose a mechanistic model linking TLR7 deficiency to epileptogenesis via two convergent pathways: a) Failure of ERV suppression. Insufficient TLR7 signalling could permit ERV reactivation in microglia or neurons. Accumulation of ERV-derived ssRNA may subsequently activate alternative innate sensors (TLR3, TLR9, RIG-I/MDA5), amplifying neuroinflammation, enhancing glutamatergic excitability, and lowering seizure threshold. b) Impaired B-cell-mediated immune regulation. Because TLR7 is essential for optimal B cell activation and antibody memory, TLR7 deficiency may diminish regulatory antibody pools needed to resolve sterile or viral inflammatory events in the CNS. This could prolong microglial reactivity and destabilise hippocampal circuits, analogous to the antibody deficiencies observed in subsets of TLE patients (Geng et al., 2019).

This framework remains speculative but testable. Targeted investigations—sequencing TLR7 in TLE cohorts, quantifying ERV expression in resected hippocampal tissue, and evaluating TLR7-knockout mice in pilocarpine and kainate models—could establish whether the TLR7–ERV axis represents a previously unrecognised immune determinant of epileptogenesis.

Neuroimaging innovations—such as TSPO-PET and emerging tracers for IL-1β, HMGB1/TLR4, and NLRP3—can map inflammatory signatures *in vivo* and guide personalised intervention (Brackhan et al., 2016). Concurrently, bioengineered platforms, including 3D brain-on-a-chip systems, humanised microglia-integrated organoids, and vascularised neural constructs, provide mechanistic insights with clinical relevance (Wu et al., 2025). These systems enable direct measurement of BBB integrity, cytokine gradients, synaptic remodelling, and immune–neural coupling.

Computational models integrating electrophysiology, spatial transcriptomics, and proteomics enable the simulation of neuroimmune feedback loops and the prediction of epileptogenic trajectories (Kumar et al., 2022). AI-driven multimodal inference may soon identify inflammatory states predictive of pharmacoresistance or epileptogenic conversion.

Near-term translational opportunities centre on deploying immune-targeted interventions in precisely defined clinical windows. Early IL-1R1 blockade in selected high-risk patients following SE represents a compelling strategy to prevent maladaptive network reorganisation. Parallel efforts should prioritise Phase I/II trials of NLRP3 inhibitors in biomarker-confirmed inflammasome-active TLE, where early pathway activation can be objectively demonstrated. HMGB1/TLR4-directed therapies may be particularly relevant for patients exhibiting elevated serum HMGB1 or positive TLR4-PET signatures, providing rational entry criteria for targeted modulation of this pathogenic axis. Advances in nanocarrier-based delivery systems offer the potential to enhance CNS penetration and achieve cell-type-specific engagement of microglia, astrocytes, or endothelial cells, thereby overcoming current pharmacokinetic limitations. Finally, immune-phenotype–based stratification using cytokine panels, PET imaging, and neurophysiological correlates will be essential to identify responders, refine therapeutic timing, and ensure that precision immunotherapy can be deployed in a manner that is both mechanistically coherent and clinically impactful. These strategies require tight integration of mechanistic biomarkers, temporal precision, and a careful balance between suppressing pathological inflammation and preserving protective immune functions.

## 10 Conclusion

The field is entering a paradigm shift in which epilepsy is reconceptualised as a disorder of chronic immune imbalance rather than solely of neuronal hyperexcitability. Dissecting the cell-type-specific, stage-dependent, and genetically modulated immune mechanisms driving epileptogenesis provides a roadmap for rational intervention. The integration of immunology, systems neuroscience, bioengineering, and computational modelling paves the way for precision immunotherapy capable of altering disease course, preserving cognition, and potentially achieving remission. The future of epilepsy care lies not only in suppressing seizures but in restoring immune–neural homeostasis.
